# Time trends in home dialysis: ERA Registry Figure of the month

**DOI:** 10.1093/ckj/sfaf325

**Published:** 2025-10-23

**Authors:** Vianda S Stel, Alberto Ortiz, Anneke Kramer

**Affiliations:** ERA Registry, Department of Medical Informatics, Amsterdam UMC - Location University of Amsterdam, Amsterdam, the Netherlands; Amsterdam Public Health Research Institute, Quality of Care, Amsterdam, the Netherlands; Department of Nephrology and Hypertension, IIS-Fundacion Jimenez Diaz UAM, Madrid, Spain; Department of Medicine, Universidad Autonoma de Madrid, Madrid, Spain; ERA Registry, Department of Medical Informatics, Amsterdam UMC - Location University of Amsterdam, Amsterdam, the Netherlands; Amsterdam Public Health Research Institute, Quality of Care, Amsterdam, the Netherlands

**Figure 1:**
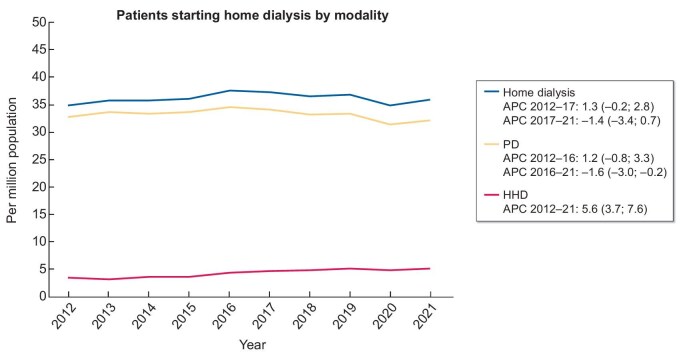
Standardized number per million population (pmp) of patients with kidney failure starting any form of home dialysis, home haemodialysis or peritoneal dialysis for the first time. **Source:** Slon-Roblero et al. NDT 2025, https://doi.org/10.1093/ndt/gfaf171, Figure 1A. **Explanation:** Most patients who started home dialysis underwent peritoneal dialysis (PD). While the home haemodialysis (HHD) initiation rate increased between 2012 and 2021, the rate for PD declined, leading to a stable overall home dialysis initiation rate throughout the study period. However, the overall incidence of both HHD and PD among kidney replacement therapy modalities remains low.

